# Outcomes of Neoadjuvant Chemoradiation With and Without Systemic Chemotherapy in Resectable and Borderline Resectable Pancreatic Adenocarcinoma

**DOI:** 10.3389/fonc.2020.01461

**Published:** 2020-09-16

**Authors:** Katherine V. Trinh, Dawn A. Fischer, Timothy B. Gardner, Kerrington D. Smith

**Affiliations:** ^1^Department of Gastroenterology and Hepatology, Dartmouth–Hitchcock Medical Center, Lebanon, NH, United States; ^2^Department of General Surgery, Dartmouth–Hitchcock Medical Center, Lebanon, NH, United States

**Keywords:** pancreas—adenocarcinoma, borderline resectable, neoadjuavant chemotherapy, chemoradiation (CRT), pancreatic cancer, systemic chemotherapy

## Abstract

**Introduction:** Neoadjuvant therapy is increasingly being used for localized pancreatic adenocarcinoma. While there is evidence supporting neoadjuvant systemic chemotherapy as well as chemoradiation, more evidence is needed to determine whether systemic chemotherapy with chemoradiation offers benefits over chemoradiation alone. This study compares the outcomes of neoadjuvant chemoradiation therapy with and without systemic chemotherapy in resectable and borderline resectable pancreatic cancers.

**Methods:** This retrospective study evaluated patients with resectable and borderline resectable pancreatic adenocarcinoma who completed neoadjuvant chemoradiation therapy with and without systemic chemotherapy prior to surgical resection. 149 patients met inclusion criteria, with 75 having resectable cancer and 74 having borderline resectable cancer. Outcomes included recurrence free and overall survival rates at 6, 12, and 36 months.

**Results:** In resectable pancreatic carcinoma, 72% of patients treated with chemoradiation alone achieved 1 year recurrence free survival compared to 78% of patients treated with systemic chemotherapy and chemoradiation (*p* = 0.55). 28% of patients treated with chemoradiation alone had 3 years recurrence free survival compared to 31% of patients who received systemic and chemoradiation therapy (*p* = 0.75). In both treatment groups, 92% of patients lived past 1 year (*p* = 0.92), and 44% of patients survived at least 3 years (*p* = 0.95). In borderline resectable pancreatic carcinoma, 50% of patients treated with chemoradiation alone achieved 1 year recurrence free survival compared to 70% of patients treated with systemic chemotherapy and chemoradiation (*p* = 0.079). The 3 years recurrence free survival was 26 and 29% for the chemoradiation alone group and the systemic chemotherapy plus chemoradiation group, respectively (*p* = 0.85). There was no significant difference in 1 year overall survival: 85% of patients treated with chemoradiation alone survived compared to 92% of patients treated with systemic chemotherapy and chemoradiation (*p* = 0.32). Both groups had 41% 3 years overall survival (*p* = 0.96).

**Discussion:** In resectable and borderline resectable pancreatic adenocarcinoma, there was no significant difference in overall or recurrence free survival between patients treated with chemoradiation with and without systemic chemotherapy. Our findings suggest that systemic neoadjuvant chemotherapy with chemoradiation and chemoradiation alone are efficacious treatments for localized pancreatic carcinoma. This brings into question whether more effective systemic chemotherapy is necessary to increase survival benefit.

## Introduction

Pancreatic cancer is the tenth most commonly diagnosed malignancy and fourth leading cause of cancer related mortality in the United States (1). Surgical resection has been considered the only curative treatment modality, however treatment options include surgical resection alone, surgical resection followed by adjuvant therapy, and neoadjuvant therapy prior to surgical resection with or without adjuvant therapy. The neoadjuvant approach has gained increasing popularity in the last decade despite controversy over its risks and benefits. Neoadjuvant therapy offers the theoretical advantages of down-staging borderline resectable or locally advanced tumors, enabling more patients to be candidates for surgical resection, increasing the rate of margin negative resection, treating occult micrometastatic disease, optimizing selection of surgical candidates, and increasing overall survival (2–4). Conversely, neoadjuvant therapy in pancreatic adenocarcinoma has variable response rates and delaying surgical resection may risk missing the opportunity for the only potentially curative modality (4, 5)

Standard definitions for resectability designation were created in an effort to standardize patients into groups based on likelihood and potential for margin negative resection. The AHPBA/SSAT/SSO guideline uses objective CT imaging criteria to designate pancreatic adenomas as resectable, borderline resectable, locally advanced, and metastatic (6). In addition to defining the role of neoadjuvant therapy in resectable and borderline resectable pancreatic cancer, more data is needed to compare different neoadjuvant regimens, including the use of systemic chemotherapy (SCT), chemoradiation therapy (CRT), or both. Several studies have demonstrated decreased rates of local recurrence and improved survival outcomes using various regimens in the preoperative setting, however more evidence is needed to elucidate the optimal treatment protocol (7, 8).

Greer et al. demonstrated that neoadjuvant gemcitabine-based CRT decreased rates of local recurrence in pancreatic adenocarcinoma (9). The PRODIGE 4/ACCORD 11 and MPACT trials demonstrated improved survival outcomes in patients with metastatic disease using neoadjuvant SCT with FOLFIRINOX and gemcitabine/abraxane over gemcitabine alone. It is still unknown whether similar results are observed in localized, non-metastatic pancreatic adenocarcinomas (10, 11). A recent study by Jang et al. found that gemcitabine based neoadjuvant CRT offered survival benefits over up front surgery in patients with borderline resectable pancreatic adenocarcinoma. This clinical trial not only demonstrated the efficacy of neoadjuvant therapy in borderline resectable disease, but also the efficacy of gemcitabine-based CRT in the absence of SCT (12).

Thus, while there is data supporting both SCT with and without CRT, as well as CRT alone, more evidence is needed to determine what the optimal neoadjuvant protocol entails in the setting of resectable and borderline resectable cancer. In this study, we compiled 13 years of data to compare the outcomes of neoadjuvant CRT with and without SCT in resectable and borderline resectable pancreatic adenocarcinoma. In addition to the previous most commonly used regimen of gemcitabine-based CRT alone and the currently popular systemic regimens of FOLFIRINOX and gemcitabine/abraxane, this retrospective study includes our institution's experience with two other neoadjuvant protocols: radio-sensitizing gemcitabine/cetuximab and systemic gemcitabine/docetaxel.

## Methods

### Study Design, Patient Selection

This study was approved by the Dartmouth Committee for the Protection of Human Subjects. We performed a single-institution retrospective study on patients at Dartmouth-Hitchcock Medical Center from 2004 to 2017. Selection criteria included patients who were reviewed by our institution's multidisciplinary gastrointestinal tumor board with histologic diagnoses of pancreatic adenocarcinoma and imaging for resectability designation. All CT scan images were reviewed retrospectively and categorized according to the AHPBA/SSAT/SSO resectability designation. Only cancers with resectable or borderline resectable designations were included in our study. Resectable tumors were defined as a primary tumor with an intact tissue plane (no venous or atrial abutment) between the tumor and the superior mesenteric vein (SMV), portal vein (PV), superior mesenteric artery (SMA), and common hepatic artery. Borderline resectable tumors were defined as radiographic evidence of tumor-associated deformity of the SMV or PV, abutment of the SMV or PV > 180°, short-segment occlusion of the SMV or PV amenable to resection and reconstruction, short-segment involvement of the hepatic artery or its branches amenable to resection and reconstruction, or abutment of the SMA <180° (6).

### Neoadjuvant Protocols

Our two treatment groups were composed of patients who received neoadjuvant radiation-sensitizing CRT alone and patients who received neoadjuvant SCT and CRT. The CRT only regimens included intensity modulated radiation therapy (IMRT) with concurrent gemcitabine alone and gemcitabine/cetuximab. The gemcitabine alone protocol consisted of 50 mg/m^2^ of gemcitabine with radiation therapy twice weekly for 12 doses with a total dose of 50.4 Gray. The gemcitabine/cetuximab protocol consisted of a loading dose of 400 mg/m^2^ cetuximab intravenously 1 week prior to radiation followed by 250 mg/m^2^ cetuximab intravenously once a week for 6 weeks in addition to 50 mg/m^2^ of gemcitabine with radiation therapy twice weekly with a total dose of 50.4 Gray.

The SCT regimens included gemcitabine/docetaxel, gemcitabine/abraxane, and FOLFIRINOX, which were sequenced upfront at the time of diagnosis prior to CRT. The gemcitabine/docetaxel regimen included 65 mg/m^2^ docetaxel and 400 mg/m^2^ gemcitabine intravenously every two weeks for three doses. The gemcitabine/abraxane regimen consisted of 1,000 mg/m2 gemcitabine and 125 mg/m^2^ abraxane weekly. Patients underwent 2–3 cycles over a period of 6–12 weeks (average 9 weeks) depending on patient response. FOLFIRINOX included biweekly 85 mg/m^2^ oxaliplatin, 400 mg/m^2^ leucovorin, 180 mg/m^2^ irinotecan, and 400 mg/m^2^ fluorouracil bolus followed by 2,400 mg/m^2^ fluorouracil via continuous infusion over 26 h. Patients underwent 4–5 cycles over a period of 8–20 weeks (average 12 weeks) depending on patient response. All SCT protocols included subsequent CRT with 50 mg/m^2^ of gemcitabine with radiation therapy twice weekly for 12 doses, with a total IMRT dose of 50.4 Gray. Table 1 lists all regimens used and their classification into SCT with CRT, and radiation-sensitizing CRT alone.

**Table 1:** Classification of neoadjuvant regimens into radiation-sensitizing agents and systemic chemotherapy agents.

**RADIATION-SENSITIZING REGIMENS**	
Gemcitabine	50 mg/m^2^ of gemcitabine with radiation therapy twice weekly for 12 doses with a total of 50.4 Gy
Gemcitabine/Cetuximab	400 mg/m^2^ cetuximab loading dose 1 week prior to radiation followed by 250 mg/m^2^ cetuximab once a week for 6 doses in addition to 50 mg/m^2^ of gemcitabine with radiation therapy twice weekly for 12 doses with a total of 50.4 Gy
SYSTEMIC CHEMOTHERAPY AGENTS^*^	
Gemcitabine/Docetaxel	65 mg/m^2^ docetaxel and 400 mg/m^2^ gemcitabine every 2 weeks for 3 doses
Gemcitabine/Abraxane	1,000 mg/m^2^ gemcitabine and 125 mg/m^2^ abraxane weekly; patients underwent 2–3 cycles over the span of 6–12 weeks depending on patient response; the average duration was 9 weeks
FOLFIRINOX	Biweekly 85 mg/m^2^ oxaliplatin, 400 mg/m^2^ leucovorin, 180 mg/m^2^ irinotecan, and 400 mg/m^2^ fluorouracil bolus followed by 2,400 mg/m^2^ fluorouracil via continuous infusion over 26 h; patients underwent 4–5 cycles over the span of 8–20 weeks depending on patient response; the average duration was 12 weeks.

* *All systemic chemotherapy protocols include subsequent chemoradiation therapy with 50 mg/m^2^ of gemcitabine with intensity modulated radiation therapy twice weekly for 12 doses, with a total IMRT radiation dose of 50.4 Gy*.

### Patient and Tumor Characteristics

We obtained baseline characteristics including patient age, gender, Carbohydrate antigen 19-9 (CA 19-9) levels, endobiliary stent presence, tumor size, tumor location, T classification, and N classification. We also collected data on neoadjuvant regimen and completion rate. Information on whether patients made it to surgical resection, whether surgical resection was completed or aborted, and whether patients received adjuvant therapy following surgical resection was also obtained.

### Outcome Measures

Intention to treat survival analysis was done on all patients who underwent neoadjuvant therapy, regardless of whether they completed their regimen or made it to surgical resection. A separate analysis included only patients who completed neoadjuvant therapy and surgical resection. We collected information on resection margins and recurrence patterns. R0 resection is defined as tumor >1 mm away from margins, and R1 resection is defined as tumor <1 mm away from margins. Local recurrence was defined as any soft tissue density that was new or increased in size compared to prior images within the resection bed, regional lymph nodes, or at the pancreaticojejunostomy or biliary anastomoses. Distant metastatic recurrence was defined as new masses outside of the surgical bed, regional draining lymph nodes, or anastomoses. Distant metastases were categorized by number and organ site. Overall survival and recurrence free survival data were collected for 6 months, 1 year, and 3 years. Both overall and recurrence free survival were calculated from date of diagnosis.

### Statistical Analysis

Continuous variables representing patient baseline characteristics and outcomes were represented as the mean and standard deviation. Categorical data were summarized as frequency and percentage. Continuous variables were analyzed using the Student's *t*-test, and categorical variables were analyzed using the chi-squared test and Fisher's exact test when appropriate. The Kaplan-Meier method was used for survival function and compared using the log rank test. The *p*-value for statistical significance was defined as ≤0.05. Statistical analysis was performed using Microsoft Excel (Microsoft Corporation, Redmond, WA) and IBM SPSS Statistics for Windows, version 23 (IBM Corporation, Armonk, NY).

## Results

The Dartmouth-Hitchcock interdisciplinary gastrointestinal tumor board saw 1,020 patients with pancreatic adenocarcinoma between 2004 and 2017. Of those cases, 742 had imaging available for retrospective AHPBA/SSAT/SSO resectability designation. Two hundred eleven patients had resectable pancreatic cancer, and 126 of those patients initiated neoadjuvant therapy. Of those 126 patients with resectable pancreatic adenocarcinoma who initiated neoadjuvant therapy, 108 patients completed treatment (86%). Seventy-five of those patients underwent surgical exploration, with 100% completing surgical resection. Of those 75 patients, 39 received preoperative CRT only and 36 completed SCT and CRT in the preoperative setting. Of the 39 patients who underwent preoperative CRT, 11 were treated with gemcitabine alone and 28 were treated with gemcitabine plus cetuximab. Of the 36 patients who received SCT and CRT, 29 received gemcitabine plus docetaxel, six received gemcitabine/abraxane, and one received FOLFIRINOX.

**Figure 1:**
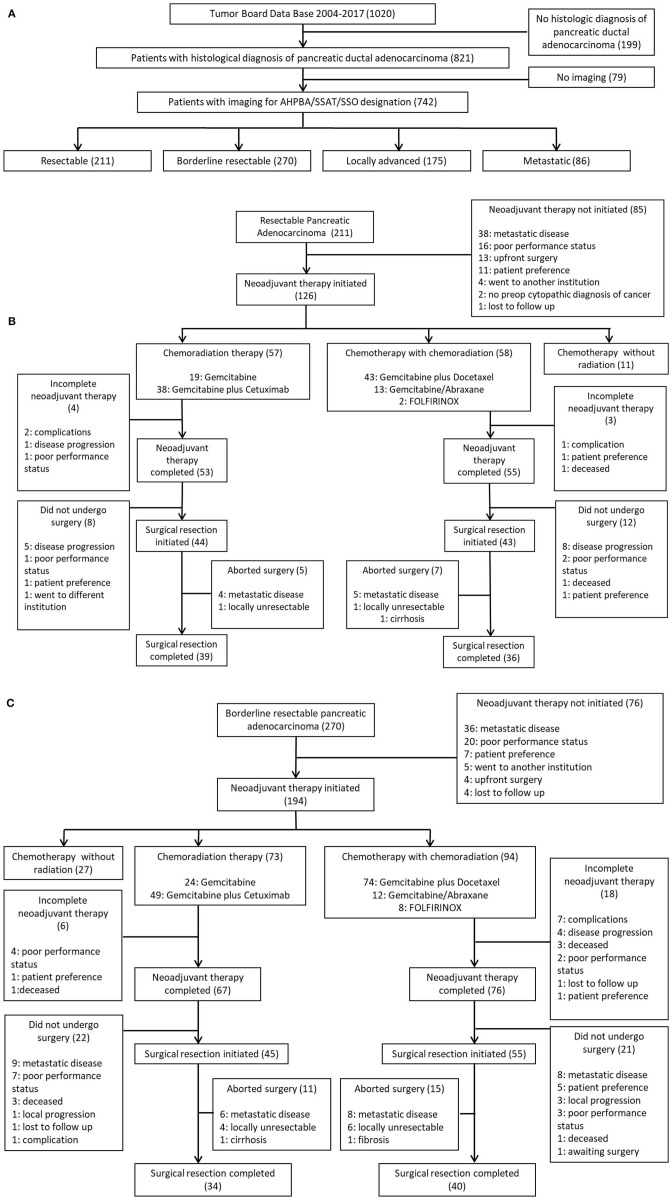
**(A)** Initial patient selection through Tumor Board and AHPBA/SSAT/SSO designation. **(B)** Progression of patients with resectable pancreatic Adenocarcinoma through neoadjuvant therapy and surgical resection. **(C)** Progression of patients with borderline resectable pancreatic adenocarcinoma through neoadjuvant therapy and surgical resection.

The borderline resectable pancreatic adenocarcinoma group consisted of 270 patients. One hundred ninety-four of those patients initiated neoadjuvant therapy. One hundred and forty-three patients out of the 194 patients who initiated neoadjuvant therapy completed treatment (74%). Hundred patients underwent surgical exploration, and 74 of those patients completed surgical resection (74%). Of those 74 patients who completed NAT and surgical resection, 34 patients received CRT alone and 40 completed SCT with CRT. Of the 34 patients who underwent preoperative CRT, six were treated with gemcitabine alone, and 28 were treated with gemcitabine plus cetuximab. Of the 40 patients who underwent SCT and CRT, 29 received gemcitabine plus docetaxel, nine received gemcitabine/abraxane, and two received FOLFIRINOX. Figure 1 maps the inclusion and progression of patients in this study. Figure 1A shows initial patient selection through Tumor Board and AHPBA/SSAT/SSO designation. Figure 1B outlines the progression of patients with resectable pancreatic adenocarcinoma through neoadjuvant therapy and surgical resection, and Figure 1C charts the progression of patients with borderline resectable pancreatic adenocarcinoma through neoadjuvant therapy and surgical resection. The follow up period for this study was 3 years, starting from date of diagnosis.

There was no difference in age, gender, endobiliary stent presence, tumor location, tumor diameter, T classification, N classification, or CA 19-9 between the two treatment groups in resectable or borderline resectable disease. These baseline characteristics are displayed in Table 2 for resectable pancreatic adenocarcinoma and Table 3 for borderline resectable cancer. There was no significant difference in adjuvant therapy treatment following surgical resection in the two treatment groups for patients with resectable disease: 28% of patients who received CRT without SCT also received adjuvant therapy compared to 33% in patients who received neoadjuvant SCT with CRT (*p* = 0.65). There was no significant difference in patients who received adjuvant therapy following resection in borderline resectable cancer, with 22 and 34% in the CRT alone and SCT with CRT treatment groups, respectively (*p* = 0.26).

**Table 2:** Baseline characteristics for patients with resectable pancreatic adenocarcinoma.

**Variable**	**Chemoradiation *n* = 39**	**Chemotherapy & chemoradiation *n* = 36**	***p*-value**
Age at diagnosis, mean (SD), years	66 (10.2)	67.2 (9.2)	0.59
**GENDER (%)**			
Female	18 (46)	13 (36)	0.38
Male	21 (54)	23 (64)	
**ENDOBILIARY STENT (%)**			
Yes	28 (72)	20 (56)	0.14
No	11 (28)	16 (44)	
**TUMOR LOCATION (%)**			
Head	30 (77)	26 (72)	0.64
Neck, body, or tail	9 (23)	10 (28)	
Tumor diameter, mean (SD), cm	2.76 (1.01)	2.68 (0.75)	0.71
CA 19-9, mean (SD)	298 (673)	571 (1,393)	0.29
**T CLASSIFICATION (%)**			
T0	2 (5)	2 (6)	0.79
T1	6 (15)	7 (19)	
T2	3 (8)	1 (3)	
T3	28 (72)	26 (72)	
**N CLASSIFICATION (%)**			
N0	31 (79)	29 (81)	0.57
N1	8 (21)	7 (19)	

**Table 3:** Baseline characteristics for patients with borderline resectable pancreatic adenocarcinoma.

**Variable**	**Chemoradiation *n* = 34**	**Chemotherapy & chemoradiation *n* = 40**	***p*-value**
Age at diagnosis, mean (SD), years	62.7 (8.4)	63.8 (8.5)	0.56
**GENDER (%)**			
Female	16 (47)	20 (50)	0.80
Male	18 (53)	20 (50)	
**ENDOBILIARY STENT (%)**			
Yes	28 (82)	29 (72)	0.32
No	6 (18)	11 (28)	
**TUMOR LOCATION, (%)**			
Head	31 (91)	31 (78)	0.11
Neck, body, or tail	3 (9)	9 (22)	
Tumor diameter, mean (SD), cm	3.97 (4.34)	3.25 (1.61)	0.33
CA 19-9, mean (SD)	576 (1,738)	443 (713)	0.69
**T CLASSIFICATION (%)**			
T0	3 (9)	0 (0)	0.057
T1	5 (15)	1 (3)	
T2	0 (0)	2 (5)	
T3	24 (71)	34 (85)	
T4	2 (6)	3 (8)	
**N CLASSIFICATION (%)**			
N0	27 (79)	26 (65)	0.13
N1	7 (21)	14 (35)	

In the resectable cancers, 74% of patients who received only CRT had R0 margins compared to 78% in the SCT with CRT group (*p* = 0.73). For both treatment groups, 92% of patents lived past 1 year (*p* = 0.92). There was no significant difference in overall 3 years survival: 46% of patients who received CRT alone survived at least 3 years compared to 44% in the SCT with CRT treatment group (*p* = 0.86). The recurrence free survival rates did not differ significantly between the treatment groups. At 1 year, 74% of patients achieved recurrence free survival in the CRT alone group compared to 75% in patients treated with SCT and CRT (*p* = 0.95). 29% of patients had recurrence free survival at 3 years in the CRT alone group compared to 25% in patients who received SCT with CRT (*p* = 0.71). The resection and survival outcomes for resectable pancreatic adenocarcinoma are displayed in Table 4.

**Table 4:** Resection and survival outcomes in patients with resectable pancreatic adenocarcinoma.

**Outcome**	**Chemoradiation**	**Chemotherapy & chemoradiation**	***p*-value**
**RESECTION MARGINS (%)**, ***n*** **=** **75**			
R0	29 (74)	28 (78)	0.73
R1	10 (26)	8 (22)	
**OVERALL SURVIVAL AT 6 MONTHS (%)**, ***n*** **=** **75**			
Yes	39 (100)	36 (100)	1.00
No	0 (0)	0 (0)	
**OVERALL SURVIVAL AT 1 YEAR (%)**, ***n*** **=** **75**			
Yes	36 (92)	33 (92)	0.92
No	3 (8)	3 (8)	
**OVERALL SURVIVAL AT 3 YEAR (%)**, ***n*** **=** **69**			
Yes	17 (46)	14 (44)	0.86
No	20 (54)	18 (56)	
**RECURRENCE FREE SURVIVAL AT 6 MONTHS (%)**, ***n*** **=** **75**			
Yes	39 (100)	36 (100)	1.00
No	0 (0)	0 (0)	
**Recurrence Free Survival at 1 year (%)**, ***n*** **=** **75**			
Yes	29 (74)	27 (75)	0.95
No	10 (26)	9 (25)	
**RECURRENCE FREE SURVIVAL AT 3 YEARS (%)**, ***n*** **=** **70**			
Yes	11 (29)	8 (25)	0.71
No	27 (72)	24 (75)	

In borderline resectable cancers, patients who received CRT alone had 68% R0 margins compared to 57% in patients who received SCT and CRT (*p* = 0.37). At 1 year, 85% of patients survived in the CRT alone group compared to 93% in patients treated with SCT and CRT (*p* = 0.32). There was no significant difference in overall 3 years survival: 41% of patients who received only neoadjuvant CRT survived at least 3 years compared to 40% in the SCT with CRT treatment group (*p* = 0.92). 56% of patients had recurrence free survival at 1 year in the CRT alone group compared to 73% in patients who received SCT with CRT (*p* = 0.14). The 3 years recurrence free survival was 26 and 30% for the CRT alone group and the SCT plus CRT group, respectively (*p* = 0.76). The resection and survival outcomes for borderline resectable pancreatic adenocarcinoma are displayed in Table 5.

**Table 5:** Resection and survival outcomes in patients with borderline resectable pancreatic adenocarcinoma.

**Outcome**	**Chemoradiation**	**Chemotherapy & chemoradiation**	***p*-value**
**RESECTION MARGINS (%)**, ***n*** **=** **74**			
R0	23 (68)	23 (57)	0.37
R1	11 (32)	17 (43)	
**OVERALL SURVIVAL AT 6 MONTHS (%)**, ***n*** **=** **74**			
Yes	34 (100)	40 (100)	1.00
No	0 (0)	0 (0)	
**OVERALL SURVIVAL AT 1 YEAR (%)**, ***n*** **=** **74**			
Yes	29 (85)	37 (93)	0.32
No	5 (15)	3 (8)	
**OVERALL SURVIVAL AT 3 YEAR (%)**, ***n*** **=** **69**			
Yes	14 (41)	14 (40)	0.92
No	20 (59)	21 (60)	
**RECURRENCE FREE SURVIVAL AT 6 MONTHS (%)**, ***n*** **=** **74**			
Yes	33 (97)	40 (100)	0.46
No	1 (3)	0 (0)	
**RECURRENCE FREE SURVIVAL AT 1 YEAR (%)**, ***n*** **=** **74**			
Yes	19 (56)	29 (73)	0.14
No	15 (44)	11 (27)	
**RECURRENCE FREE SURVIVAL AT 3 YEARS (%)**, ***n*****=71**			
Yes	9 (26)	11 (30)	0.76
No	25 (74)	26 (70)	

**Figure 2:**
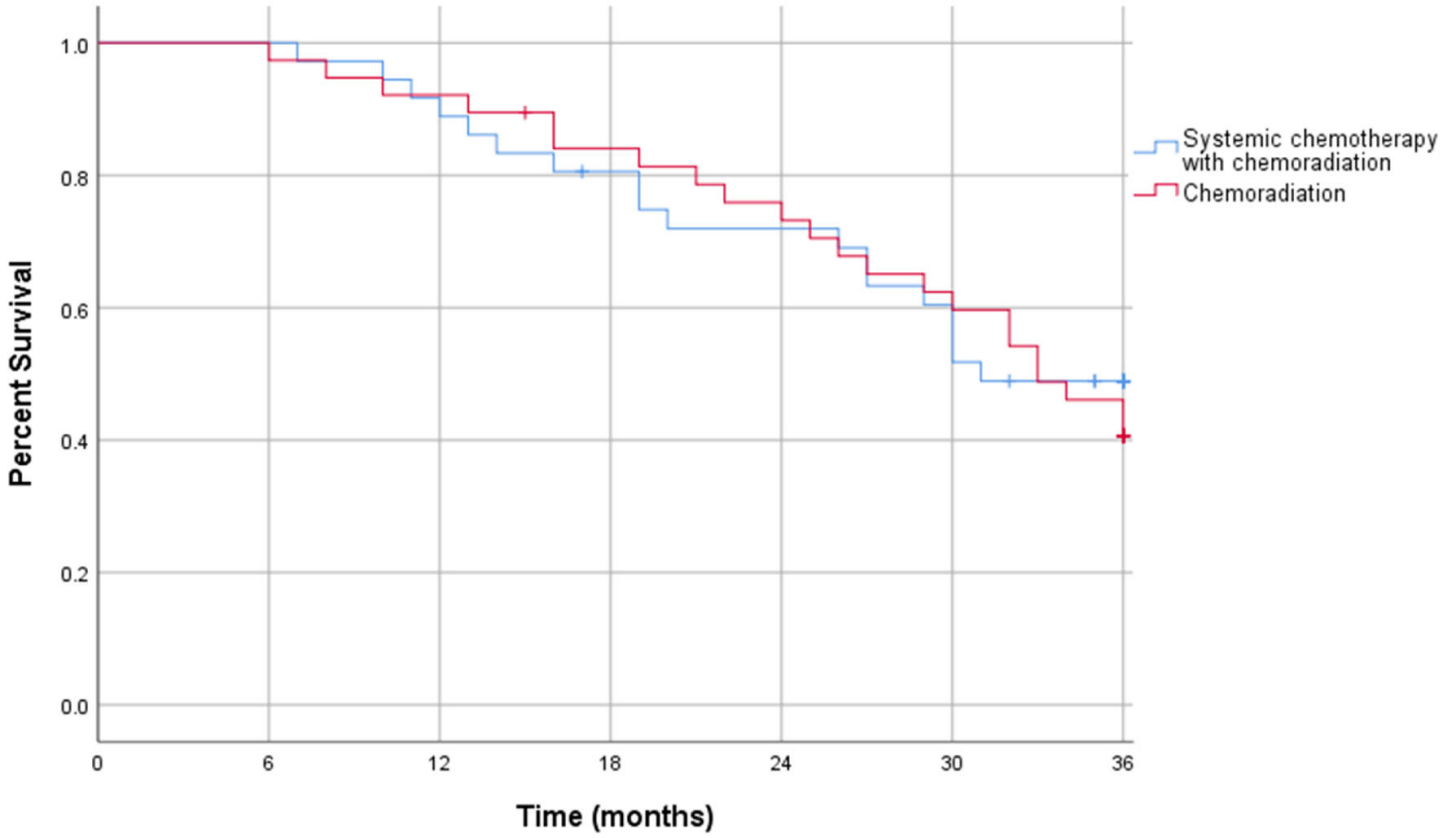
Kaplan-Meier curve for resectable pancreatic adenocarcinoma.

**Figure 3:**
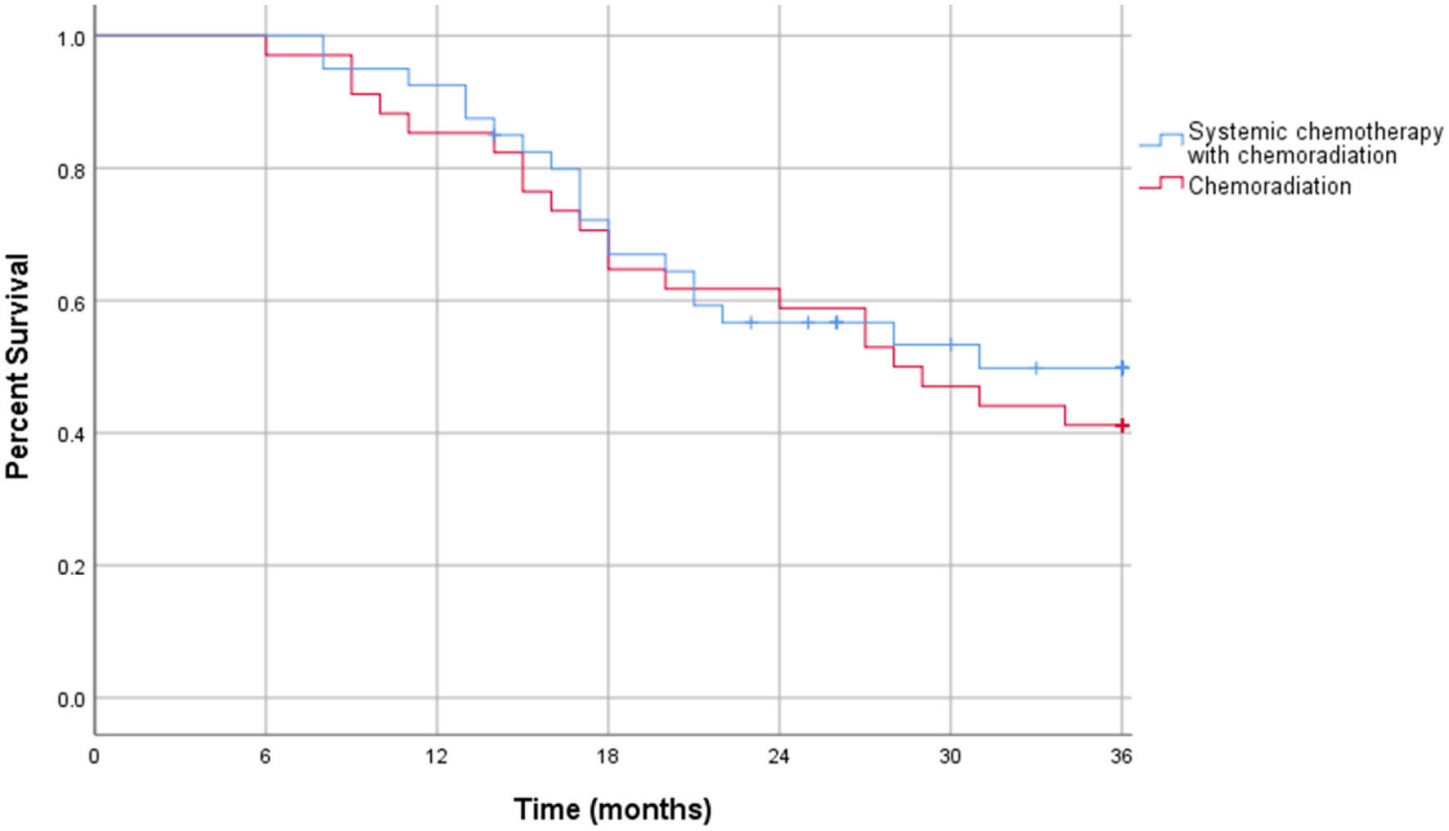
Kaplan-Meier curve for borderline resectable pancreatic adenocarcinoma.

The Kaplan-Meier curve for resectable pancreatic adenocarcinoma showed no difference in the survival function up to 3 years between the two treatment groups, with a mean survival of 28.19 months for SCT with CRT and 28.93 for CRT alone (*p* = 0.87) (Figure 2). For borderline resectable adenocarcinoma, the Kaplan-Meier curve also showed no difference in the survival function between the two treatment groups, with a mean survival of 26.83 months for SCT with CRT and 25.94 for CRT alone (*p* = 0.58) (Figure 3). There was no significant difference in recurrence patterns (local, distant, or both) between the treatment groups in either resectable or borderline resectable pancreatic adenocarcinoma (Tables 6, 7, respectively).

**Table 6:** Pattern of recurrence in patients with resectable pancreatic adenocarcinoma who completed neoadjuvant therapy and surgical resection.

**Pattern of recurrence**	**Chemoradiation**	**Chemotherapy & Chemoradiation**	***p*-value**
**ANY RECURRENCE (%)**, ***n*** **=** **75**			
Yes	27 (69)	21 (58)	0.37
No	12 (21)	15 (42)	
**LOCAL ONLY (%)**, ***n*** **=** **74**			
Yes	5 (13)	1 (3)	0.11
No	33 (87)	35 (97)	
**DISTANT ONLY (%)**, ***n*** **=** **74**			
Yes	16 (42)	16 (44)	0.84
No	22 (58)	20 (56)	
**BOTH LOCAL AND DISTANT (%)**, ***n*** **=** **74**			
Yes	5 (13)	4 (11)	0.54
No	33 (87)	32 (89)	

**Table 7:** Pattern of recurrence in patients with borderline resectable pancreatic adenocarcinoma who completed neoadjuvant therapy and surgical resection.

**Pattern of recurrence**	**Chemoradiation**	**Chemotherapy & chemoradiation**	***p*-value**
**ANY RECURRENCE (%)**, ***n*** **=** **74**			
Yes	25 (74)	30 (75)	0.89
No	9 (26)	10 (25)	
**Local ONLY (%)**, ***n*** **=** **74**			
Yes	5 (15)	1 (3)	0.067
No	29 (85)	39 (97)	
**DISTANT ONLY (%)**, ***n*** **=** **74**			
Yes	15 (44)	16 (40)	0.72
No	19 (56)	24 (60)	
**BOTH LOCAL AND DISTANT (%)**, ***n*** **=** **74**			
Yes	5 (15)	4 (10)	0.34
No	29 (8)	36 (90)	

**Figure 4:**
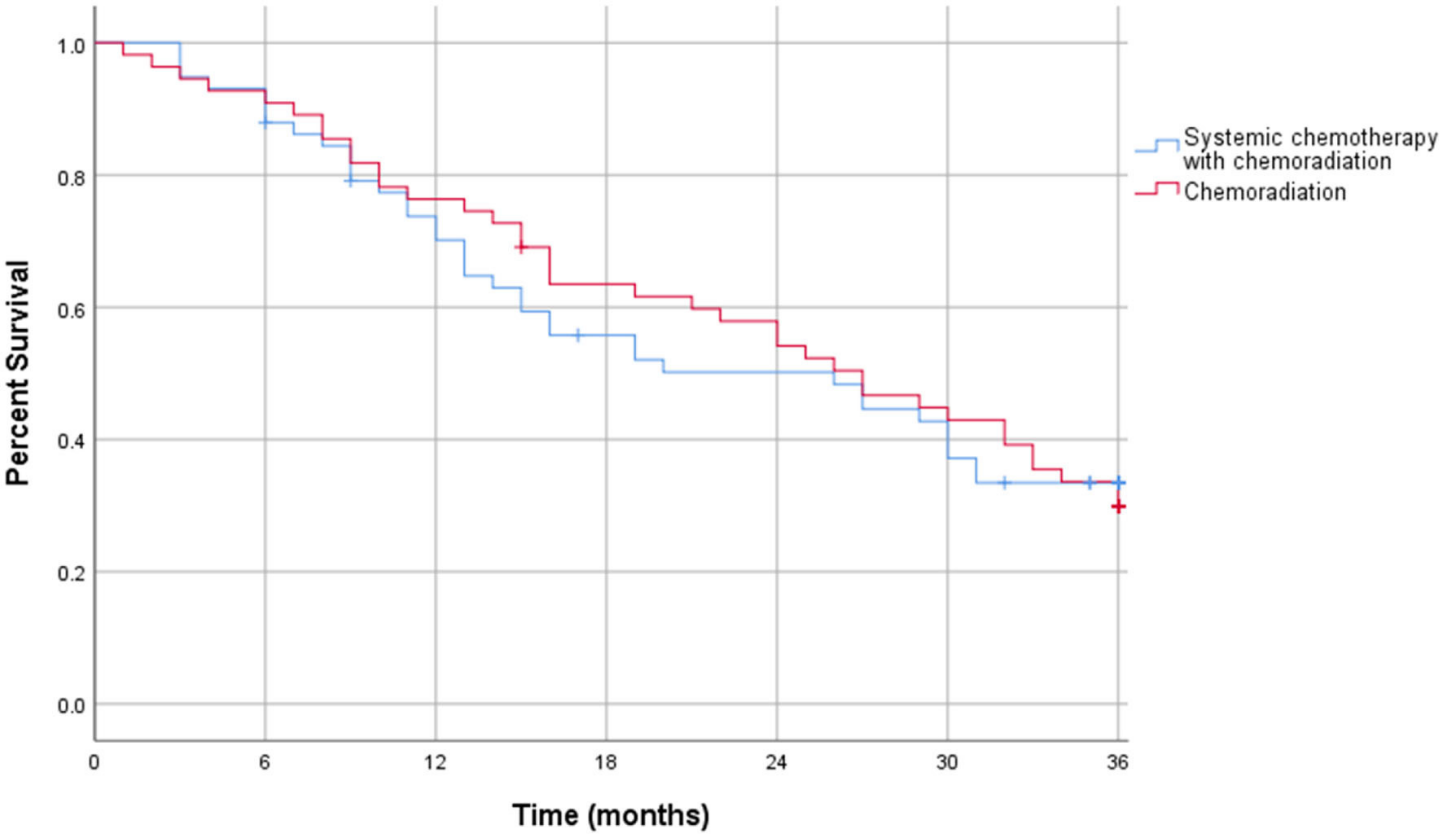
Intention to treat Kaplan-Meier curve for resectable pancreatic adenocarcinoma.

Additional survival analysis was done using intention to treat methodology and included all patients who initiated neoadjuvant therapy, regardless of neoadjuvant therapy completion or surgical resection. In the intention to treat survival analysis for resectable pancreatic adenocarcinoma, there was no significant difference in 1 year overall survival: 38% of patients who received CRT alone compared to 37% in the SCT with CRT treatment group (*p* = 0.66). There was also no difference in 3 years overall survival with 17% in CRT patients and 14% in SCT with CRT patients (*p* = 0.62). These outcomes are displayed in Table 8. The intention to treat Kaplan-Meier curve for resectable pancreatic adenocarcinoma showed no difference in the survival function up to three years between the two treatment groups, with a mean survival of 22.32 months for SCT with CRT and 23.81 for CRT alone (*p* = 0.89) (Figure 4).

**Table 8:** Intention to treat survival outcomes in patients with resectable pancreatic adenocarcinoma.

**Outcome**	**Chemoradiation**	**Chemotherapy & chemoradiation**	***p*-value**
**OVERALL SURVIVAL AT 6 MONTHS (%)**, ***n*** **=** **114**			
Yes	52 (46)	54 (47)	0.96
No	4 (4)	4 (4)	
**OVERALL SURVIVAL AT 1 YEAR (%)**, ***n*** **=** **112**			
Yes	43 (38)	41 (37)	0.66
No	13 (12)	15 (13)	
**OVERALL SURVIVAL AT 3 YEAR (%)**, ***n*** **=** **106**			
Yes	18 (17)	15 (14)	0.62
No	36 (34)	37 (35)	

**Figure 5:**
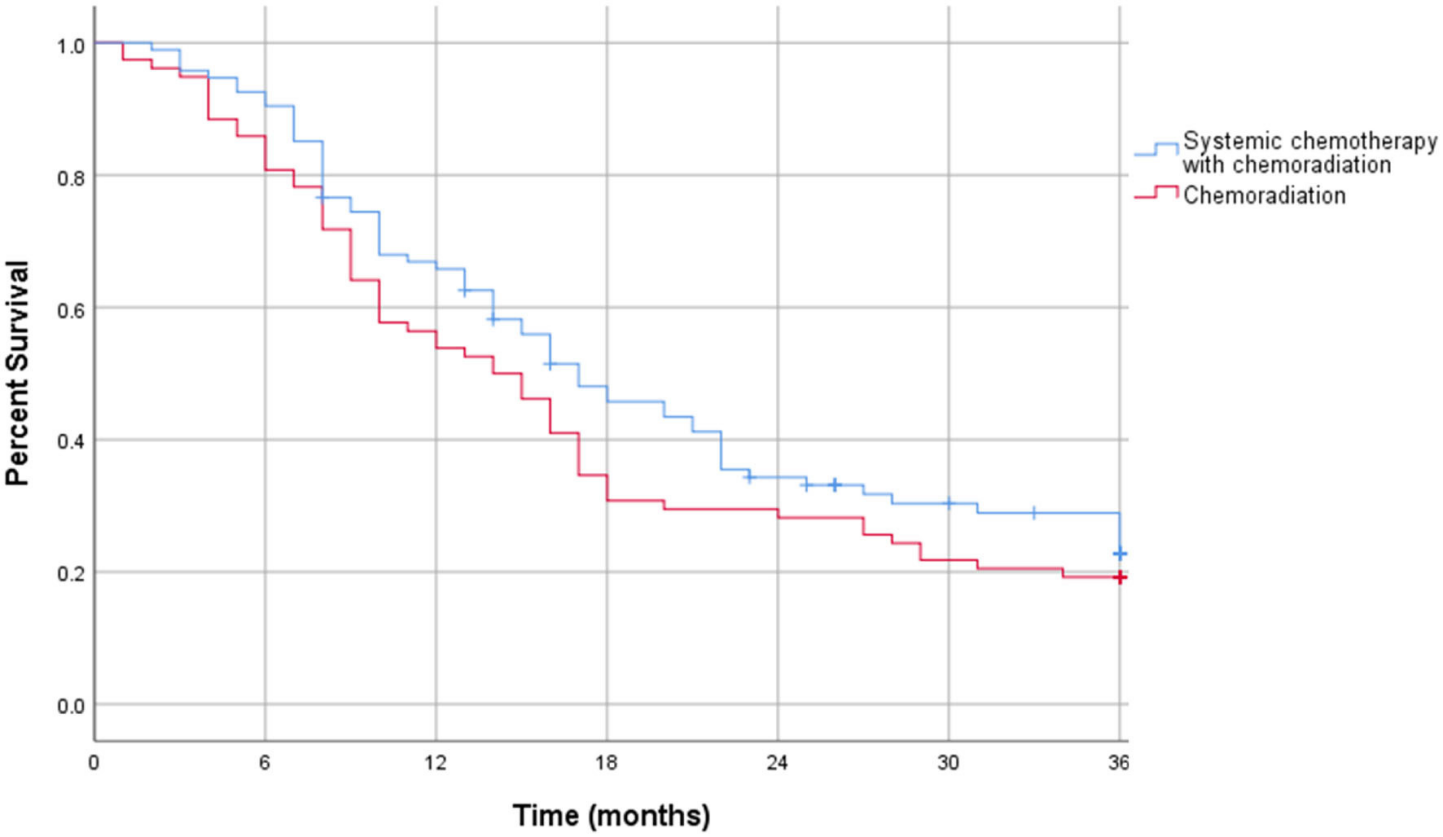
Intention to treat Kaplan-Meier curve for borderline resectable pancreatic adenocarcinoma.

The intention to treat survival analysis for borderline resectable pancreatic adenocarcinoma demonstrated no significant difference in overall all survival rate. At 1 year, 34% of patients survived in the CRT alone group compared to 36% in patients treated with SCT and CRT (p = 0.27). At 3 years post diagnosis, there was a 9% survival rate in the CRT alone group compared to 12% in patients treated with SCT and CRT (*p* = 0.53). The intention to treat survival outcomes for borderline resectable pancreatic adenocarcinoma are displayed in Table 9. Additionally, the intention to treat Kaplan-Meier curve for borderline resectable disease showed no difference in the survival function between the two treatment groups, with a mean survival of 19.99 months for SCT with CRT and 17.08 for CRT alone (*p* = 0.19) (Figure 5).

**Table 9:** Intention to treat survival outcomes in patients with borderline resectable pancreatic adenocarcinoma.

**Outcome**	**Chemoradiation**	**Chemotherapy & chemoradiation**	***p*-value**
**OVERALL SURVIVAL AT 6 MONTHS (%)**, ***n*** **=** **172**			
Yes	67 (39)	87 (51)	0.16
No	11 (6)	7 (4)	
**OVERALL SURVIVAL AT 1 YEAR (%)**, ***n*** **=** **171**			
Yes	58 (34)	62 (36)	0.27
No	20 (12)	31 (18)	
**OVERALL SURVIVAL AT 3 YEARS (%)**, ***n*** **=** **164**			
Yes	15 (9)	20 (12)	0.53
No	63 (38)	66 (40)	

## Discussion

While the efficacy of neoadjuvant therapy in localized pancreatic adenocarcinoma is still highly debated, neoadjuvant therapy is increasingly being utilized at institutions across the world. With modern systemic chemotherapy, oncologic outcomes in clinical trials show improved overall survival benefits not only in metastatic disease, but in localized cancer as well (7–9, 13, 14). In addition to defining the role of neoadjuvant therapy, more insight is needed on what the optimal treatment regimen is and whether that includes SCT, CRT, or both. The existing data comparing CRT alone and SCT plus CRT in locally advanced disease is contradictory: in comparing SCT with and without CRT, Hugeut et al. found survival benefits in patients who received SCT with CRT, while Hammal et al. saw no improvement in survival in patients who received SCT and CRT (15, 16). While multiple trials have compared different regimens and combinations of SCT, CRT, or both in resectable and borderline resectable pancreatic adenocarcinoma, there is still more work to elucidate the optimal treatment regimen (17–21).

This study reports 13 years of experience using neoadjuvant therapy for pancreatic adenocarcinoma with regimens including SCT with CRT, and CRT alone. The majority of patients with resectable and borderline pancreatic carcinoma who initiated neoadjuvant therapy completed their regimens and made it to surgical resection. We compared margin negative resection rates as well as recurrence patterns and saw no difference between the two treatment groups in either resectable or borderline resectable disease. We also did not find any significant advantage in overall or recurrence free survival between patients treated with SCT plus CRT vs. CRT alone. Our intention to treat survival analysis was consistent with our findings from examining only patients who achieved surgical resection.

Our findings suggest that for resectable and borderline resectable pancreatic adenocarcinoma, SCT with CRT does not offer any survival or recurrence benefits compared to CRT alone. This brings into question whether neoadjuvant SCT is necessary if neoadjuvant CRT alone is just as effective. SCT with CRT not only increases the delay in surgical resection compared to CRT alone, but it also subjects patients to increased side effects and drug toxicity (4). An alternative treatment approach to consider is using neoadjuvant CRT followed by surgical resection and systemic adjuvant therapy. This approach may provide patients with the benefits of neoadjuvant therapy, while decreasing further delay in potentially curative resection and offering the benefits of systemic therapy. A clinical trial is needed to provide insight on whether SCT in the neoadjuvant or adjuvant setting provides more benefits in patients receiving neoadjuvant CRT with resectable and borderline resectable pancreatic adenocarcinoma.

This study has several limitations. First, it is limited by its sample size and non-randomized observational design. The type of neoadjuvant therapy a patient received was made on an individual basis by the treating physicians. There was no over-arching guide, and it is possible that patients with more adverse clinical features were preferentially given one type of therapy over another, as this was not controlled for in our analysis. Additionally, grouping the different regimens into SCT with CRT vs. CRT alone does not allow us to compare each individual regimen directly. The heterogeneity in the SCT with CRT group includes the currently popular systemic regimens of FOLFIRINOX and gemcitabine/abraxane as well as old regimens no longer commonly used. This may account for the lack of benefit seen over CRT alone, as recent studies focusing on these newer regimens have shown promising results in borderline and locally advanced disease, and trials in resectable disease are beginning to shed more light (22–25). Although intention to treat survival analysis was done, some of our other data analysis excluded patients who were not surgically resected. This number was similar in both treatment groups for both resectable and borderline resectable cancer however, and was done to allow analysis of variables such as resection margins and recurrence patterns. Lastly, the follow up time of this study limits the detection of a difference in the overall and recurrence free survival beyond 3 years.

In conclusion, both SCT with CRT and CRT alone are effective options for neoadjuvant therapy in resectable and borderline resectable pancreatic adenocarcinoma. Our findings bring into question whether SCT is necessary to increase survival benefits if neoadjuvant CRT alone is just as effective. This argues for the need for better systemic therapy to justify its use, or whether neoadjuvant CRT alone should be used with adjuvant SCT following resection. While there is need for a prospective randomized clinical trial, our study is an early attempt at elucidating the effects of neoadjuvant SCT with CRT compared to CRT alone in resectable and borderline resectable pancreatic adenocarcinoma.

## Data Availability Statement

The raw data supporting the conclusions of this article will be made available by the authors, without undue reservation, to any qualified researcher.

## Ethics Statement

The studies involving human participants were reviewed and approved by Dartmouth Committee for the Protection of Human Subjects. The patients/participants provided their written informed consent to participate in this study.

## Author Contributions

KT, DF, TG, and KS contributed to the study concept, design, acquisition of data, analysis, interpretation, drafting, and editing of the manuscript, and critical revision. All authors contributed to the article and approved the submitted version.

## Conflict of Interest

The authors declare that the research was conducted in the absence of any commercial or financial relationships that could be construed as a potential conflict of interest.
